# Microbial reduction of organosulfur compounds at cathodes in bioelectrochemical systems

**DOI:** 10.1016/j.ese.2020.100009

**Published:** 2020-01-07

**Authors:** Margo Elzinga, Dandan Liu, Johannes B.M. Klok, Pawel Roman, Cees J.N. Buisman, Annemiek ter Heijne

**Affiliations:** a Environmental Technology, Wageningen University, Bornse Weilanden 9, P.O. Box 17, 6700, AA Wageningen, the Netherlands; b Paqell B.V, Reactorweg 301, 3542, AD Utrecht, the Netherlands; c Wetsus, Centre of Excellence for Sustainable Water Technology, Oostergoweg 9, P.O. Box 1113, 8900, CC Leeuwarden, the Netherlands

**Keywords:** Thiols, Organosulfur compounds, Bioelectrochemical system

## Abstract

Organosulfur compounds, present in e.g. the pulp and paper industry, biogas and natural gas, need to be removed as they potentially affect human health and harm the environment. The treatment of organosulfur compounds is a challenge, as an economically feasible technology is lacking. In this study, we demonstrate that organosulfur compounds can be degraded to sulfide in bioelectrochemical systems (BESs). Methanethiol, ethanethiol, propanethiol and dimethyl disulfide were supplied separately to the biocathodes of BESs, which were controlled at a constant current density of 2 A/m^2^ and 4 A/m^2^. The decrease of methanethiol in the gas phase was correlated to the increase of dissolved sulfide in the liquid phase. A sulfur recovery, as sulfide, of 64% was found over 5 days with an addition of 0.1 ​mM methanethiol. Sulfur recoveries over 22 days with a total organosulfur compound addition of 1.85 ​mM were 18% for methanethiol and ethanethiol, 17% for propanethiol and 22% for dimethyl disulfide. No sulfide was formed in electrochemical nor biological control experiments, demonstrating that both current and microorganisms are required for the conversion of organosulfur compounds. This new application of BES for degradation of organosulfur components may unlock alternative strategies for the abatement of anthropogenic organosulfur emissions.

## Highlights

• Organosulfur compounds can be reduced to sulfide at biocathodes.
• Biological conversions of OSC are possible in a current controlled environment.
• Organosulfur compounds can be reduced by microbial species from various environments.
• Bioelectrochemical systems may form a new technology for industrial removal of OSC.

## Graphical abstract

**Figure:**
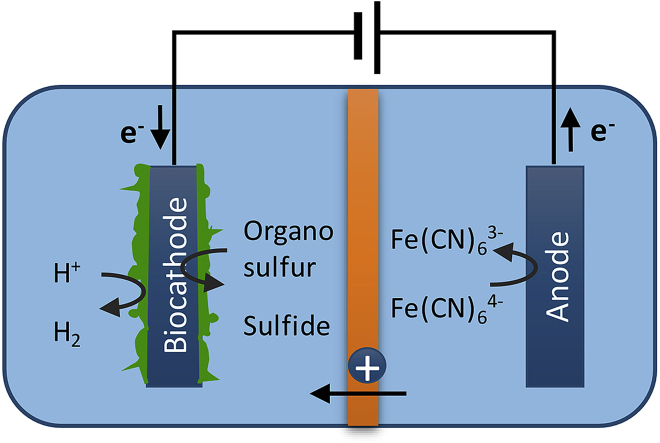

## 1 Introduction

Organosulfur compounds (OSCs) are naturally present in various environments, including oceans, marine estuaries, volcanos and salt marshes. These organsulfur compounds play an important role in the natural global sulfur cycle [1,2]. However, anthropogenic emissions of e.g. methanethiol and dimethyl disulfide account for 30% of the annual global emissions with 3222 GgS/year. Methanethiol, dimethyl disulfide and other compounds like ethanethiol and propanethiol are found in the pulp and paper industry, rayon and cellulose industry and (bio)gas streams [1]. Removal of organosulfur compounds from waste streams is required due to the low odor thresholds, high toxicity and their corrosive nature.

The state-of-the-art treatment strategy for conversion and removal of organosulfur compounds is the oxidation to insoluble disulfides. This process, known as the Merox process, is in many cases economically unfavorable due to complex processing schemes, high OPEX and CAPEX and low efficiencies [3]. In addition, this process requires chelating chemicals, which can harm the environment. Lacking a suitable treatment method, organosulfur compounds are typically incinerated contributing to SO_2_ emissions. The emissions of SO_2_ are strictly regulated and are becoming more stringent over the years. Therefore, new cost-effective, environmental friendly strategies for the removal of organosulfur compounds are desired.

In general, biological processes are considered environmentally friendly as they require ambient temperatures and pressures and do not require chemical catalysts. Methanethiol and dimethyl disulfide can be converted under aerobic conditions in bio trickling systems [4]. However, aerobic degradation only occurs at low concentrations and degradation rates are low. On the other hand, anaerobes, such as methanogenic archaea, are known to tolerate much higher thiol concentrations and, therefore, represent an alternative for the treatment of thiol containing waste streams. Several studies report successful biological reduction of methanethiol and dimethyl disulfide to methane and hydrogen sulfide by methylotrophic methanogens [[5], [6], [7]].

The degradation of ethanethiol and propanethiol appears to be much more challenging. Degradation in anaerobic bioreactor has, to the best of our knowledge, not been reported. Leerdam et al. was unable to convert these organosulfur compounds in anaerobic batch systems [8]. Only two studies report an enhanced production of ethane and propane in anoxic sediments when ethanethiol and propanethiol are supplied [9,10]. The conversion efficiencies were low, less than one percent of the added substrates, and Oremland et al. [9] suggested that conversions of ethanethiol were solely a result of co-metabolism and growth on ethanethiol as sole substrate was not possible. Nevertheless, these studies suggest that biological conversion mechanisms exist.

Bioelectrochemical systems (BESs) are an emerging biotechnology with a wide range of applications, e.g. electricity generation, metal recovery, chemicals synthesis, and wastewater treatment [[11], [12], [13], [14]]. In bioelectrochemical systems, microorganisms catalyze anodic oxidation reactions or cathodic reduction reactions. Reaction rates can be manipulated by controlling electrode potential or current density. Complete reduction of organosulfur compounds at a biocathode would result in the formation of methane and sulfide. These products have as advantage that in the (bio)gas industry methane can be directly used and elemental sulfur can be recovered from the hydrogen sulfide with existing technologies. In this study, we demonstrate that methanethiol, ethanethiol, propanethiol and dimethyl disulfide can be converted at biocathodes, and that degradation requires both microorganisms and electricity.

## 2 Materials and methods

### 2.1 Bioelectrochemical cell setup

Bioelectrochemical experiments were performed in 4 identical anaerobic reactors (H cells [15]). Cells consisted of two 150 ​mL chambers separated by a cation exchange membrane (8.02 ​cm^2^ CEM, fumasep®FTCM-E, Fumatech, Germany). The experiment was performed under continuous stirring at 350 RPM. Gas produced at the cathode was collected in 1 ​L gas bags (Cali-5-Bond^TM^, Calibrated Instruments Inc., USA) with an initial gas volume of 35 ​mL. Graphite felt electrodes (0.4 ​cm ​× ​2 ​cm x 15cm, CTG Carbon GmbH, Germany) connected to a platinum current collector were used as both anode and cathode electrodes. A 3 ​M KCl Ag/AgCl reference electrode (+210mV vs SHE, Prosense, Oosterhout, the Netherlands) was inserted in the cathode chamber. The current was controlled with a potentiostat (Ivium, the Netherlands). Cells were operated at room temperature (22–25 ​°C).

### 2.2 Medium and inoculum

A bicarbonate medium similar to the medium used for biodesulfurization processes under haloalkaline conditions [16] was used during all experiments. The medium contained 49 ​g/L NaHCO_3_, 4.42 ​g/L Na_2_CO_3_ and 0.1 ​mL/L nutrient solution containing N, P and trace elements (Paqell B.V., The Netherlands). The medium was flushed with N_2_ for 20 ​min, resulting in a pH of 8.5. Anolyte contained the same carbonate buffer with 84.5 ​g/L potassium hexacyanoferrate(II)trihydrate. Hexacyanoferrate (II) is a typical substrate in bioelectrochemical system used as an electron donor in the anode to avoid the crossover of produced oxygen at the anode to the cathode when water is oxidized at the anode.

Cathode chambers were inoculated with a mixture of biomass selected based on their acclimation to anaerobic conditions, high salt concentrations, the presence of organosulfur compounds and the presence of methanogens. The inocula were obtained from (1) a chain elongation reactor fed with high methanol concentrations (250 ​mM)(total nitrogen (TN) 1.47 ​g/L) [17],(2) a granular anaerobic reactor operating at a high salt concentration (20 ​g Na/L) [18] (TN 4.3 ​g/L), (3) a digester for municipal wastewater treatment sludge (Ede, The Netherlands) combined with anaerobic sludge treating wastewater from paper industry (Eerbeek, The Netherlands)(4.4 ​g/L TCOD), and (4) sulfide oxidizing biomass adapted to the presence of 0.5–2.5 ​mM dimethyl disulfide (TN 0.6 ​g/L) [19]. The biomass mix was obtained by combining 2 ​mL of the the different inocula. 1 ​mL of the biomass mix was added to the cells during startup.

### 2.3 Bioelectrochemical cell operation

For initial proof-of-principle experiments, sludge from the paper industry and municipal wastewater treatment plant was used to inoculate two cells. The reactors were started with 1mM methanol (150 ​μmol) and 0.1 ​mM methanethiol (15 ​μmol) at the biocathodes and were operated for one week to stimulate initial growth and to acclimate the biomass. Subsequently, medium was replaced and methanol (150 ​μmol) and methanethiol (22.5 ​μmol to cell 1 and 15 ​μmol to cell 2) were supplied once. Cells were galvanostatically controlled at 2 ​mA (2 A/m^2^ projected surface area of the graphite felt cathode). Gas phase concentrations of organosulfur compounds and sulfide concentrations in solution were monitored for 5 days.

In the next experimental run, we studied the degradation of all four organosulfur compounds. Each organosulfur compound was supplied to a biocathode in a pre-experimental run 13 days prior to the start of the experimental run, allowing microorganisms to adapt to experimental conditions. 1 ​ml of biomass mix (see section 2.2) and 150 μmol methanol were added to all cells. Upon the detection of methane, indicating biological activity, 15 ​μmol of methanethiol, ethanethiol, propanethiol and dimethyl disulfide were added to individual cells every weekday. Cells were galvanostatically controlled at 2mA, obtaining a current density of 2 A/m^2^ normalized to the projected surface area of the graphite felt electrode. Medium from the pre-experimental run was replaced at the start of the experimental run. Suspended biomass in the medium was collected by centrifuging for 15 ​min at 5000 ​rpm and was returned to the cells. Biomass mix (1 ​mL) and methanol (150 ​μmol) were added to the cells on day 0. Cells remained under galvanostatic control (2 ​mA/m^2^) and 15 ​μmol of organosulfur compounds were added daily. After 12 days, organosulfur compound additions were doubled and current density was doubled to 4 A/m^2^. The cells were operated in this mode for 10 days.

### 2.4 Electrochemical, biotic and abiotic control experiments

Electrochemical control experiments were performed in the same electrochemical setup as described in 2.3 with a current density of 2 ​mA/m^2^. New graphite felt electrodes without microorganisms were used. Organosulfur compounds (15 ​μmol) were added individually to the cells, which were operated for 24 ​h. Additional electrochemical experiments were performed with 15 ​μmol dimethyl disulfide, 150 ​μmol methanol and biomass from the different sources added to individual cells. The cells were operated until sulfide production was shown in each of the cells.

Biotic control experiments with hydrogen as electron donor were performed in 250 ​mL serum flasks. At the start of the experiment the headspace was replaced with a CO2/H2 (80:20) gas mixture at 1.3 ​bar. Flasks contained 150 ​mL medium, methanol (150 ​μmol) and were inoculated with 1 ​mL biomass mix. The start pH was between 7.7 and 8. Upon the production of methane, each serum flask was supplied with 15 ​μmol of one single organosulfur compound. Flasks were placed in an incubator and mixed at 150 RPM at 22–25 ​°C for 30 days.

Abiotic control experiments with methanethiol were performed in 250 ​mL serum flasks under 100% nitrogen at atmospheric pressure. Individual flasks were filled with 150 ​mL medium and water and 0.1 ​mM methanethiol.

### 2.5 Analytical techniques and calculations

We used organosulfur gas phase analysis to validate the use of sulfide as indicator for organosulfur compound degradation. Gas phase concentrations of methanethiol and dimethyl disulfide were analyzed as described by Roman et al., 2015 [20]. Analysis of organosulfur compounds to show their degradation is challenging as (i) organosulfur compound analyses are prone to errors due to their volatile nature, and (ii) a decrease in organosulfur concentrations does not necessarily indicate its conversion, as it could also indicate leakage of organosulfur from the system.

Total sulfide (S^2−^, HS^-^ and H_2_S) in the liquid was measured using the methylene blue method (Hach LCK 653), in which sulfide is converted to H_2_S. To avoid measurement errors due to the alkaline nature of the samples, dilutions were performed with 0.5 ​M sulfuric acid. The method was tested for interference with organosulfur compounds by measuring solutions of sulfide with and without organosulfur compounds, and no interference was observed.

Sulfate and thiosulfate, were analyzed with Ion Chromatography (see SI-1). Gas chromatography was used to measure CH_4_, N_2_, O_2_, CO_2_ and H_2_ using the methods described by Liu et al., 2017 [21].

Next Generation Sequencing was used to analyze the microbial community. The Powersoil DNA isolation kit was used for DNA extractions. DNA amplification via PCR and sequencing were performed as described by Takahashi et al. [22].

Coulombic efficiency was calculated as

$$CE\;\left(\%\right)=\;\frac{V\times \left[OSC-S\right]\times n\times F}{\int_{t0}^{t}I\;dt}\;\times 100$$

Where V is the catholyte volume (0.15 ​L), [OSC-S] is the concentration of sulfur added (mol/L), n is the number of electrons for reduction of organosulfur compounds to CH_4_ and HS^-^ (See eq (1), (2), (3), (4))), F is the Faraday constant (96485 ​C/mol), I is current (A) and t is time (s).

(1) CH_3_SH ​+ ​H^+^ + 2e^-^ → CH_4_ ​+ ​HS^-^

(2) C_2_H_5_SH ​+ ​3H^+^ ​+ ​4e^-^ → 2CH_4_ ​+ ​HS^-^

(3) C_3_H_7_SH ​+ ​5H^+^ ​+ ​6e^-^ → 3CH_4_ ​+ ​HS^-^

(4) C_2_H_6_S_2_ ​+ ​4H^+^ ​+ ​4e^-^ → 2CH_4_ ​+ ​2HS^-^

## 3 Results and discussion

### 3.1 Methanethiol degradation in BES

**Fig. 1:**
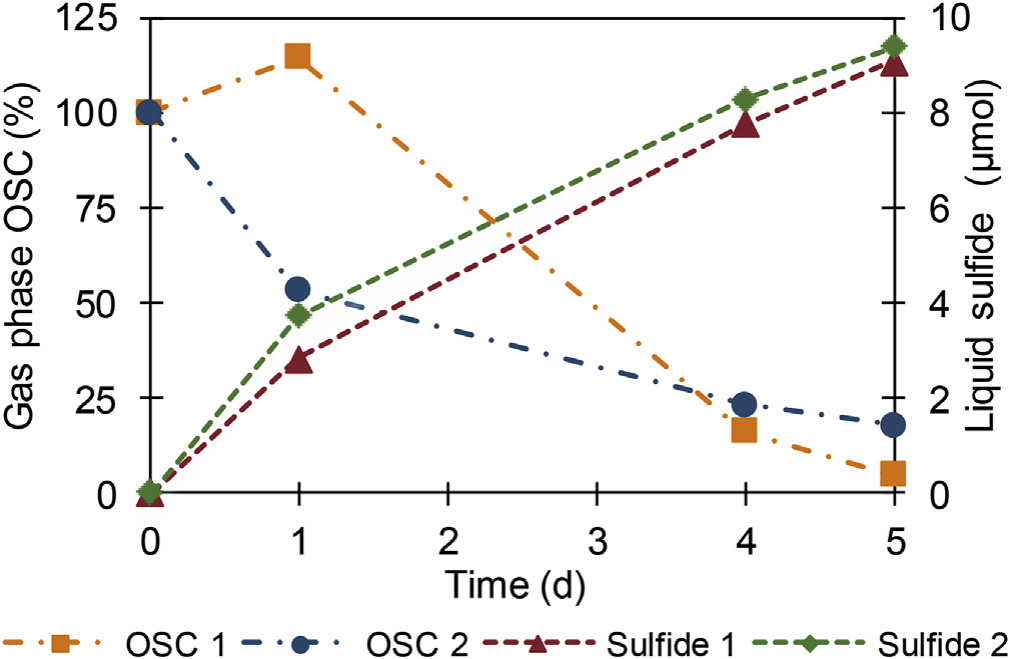
Degradation of organosulfur compounds(OSC) at biocathodes. Decrease of organosulfur compounds in the gas phase, consisting of the sum of methanethiol and its oxidation product dimethyl disulfide, was related to the increase of sulfide in the liquid phase. The organosulfur compounds are shown as a percentage of the initial absolute amount present in the gas phase.

Gaseous methanethiol and its oxidation product dimethyl disulfide, expressed as organosulfur compounds, and dissolved sulfide were analyzed over time at the biocathodes of two BES cells. Organosulfur compounds were almost completely removed from the gas phase in 5 days. The gas phase concentration decreased with 95% starting with 0.13 ​μmol in cell 1 and with 82% in cell 2 starting with 0.19 ​μmol, while the sulfide in the liquid increased from <0.5 to 9.1 ​μmol (61 ​μM) in cell 1 and to 9.3 ​μmol (62 ​μM) in cell 2 (Fig. 1). A considerable part of the supplied methanethiol was converted and recovered as sulfide. After 5 days: 41% of the 22.5 ​μmol (0.15 ​mM) methanethiol supplied in cell 1, and 64% of the 15 ​μmol (0.1 ​mM) methanethiol supplied to cell 2 was obtained as sulfide in the liquid phase. The fate of the remaining methanethiol fraction is unclear. However, there are several possible reasons why not all methanethiol was recovered as sulfide. First, a fraction of the methanethiol could be unconverted and still present in the liquid phase as there was still some methanethiol present in the gas phase. Second, methanethiol could be lost from the system through diffusion via or adsorption onto the membrane, tubing, electrodes and sampling ports. However, this was not quantified. Finally, the measured sulfide may underestimate the produced sulfide, since part of the sulfide could be used by microorganisms for growth. Abiotic controls, without electrical current, showed a minor decrease of gaseous organosulfur compounds of 1.1% in medium and 3.1% in Milli-Q (see SI-2) demonstrating that no degradation of methanethiol occurred in absence of bacteria and electrical current. Sulfide formation was used in further experiments to indicate organosulfur compound conversions, since sulfide formation provides a convincing proof that organosulfur compounds are converted.

### 3.2 Degradation of organosulfur compounds

**Fig. 2:**
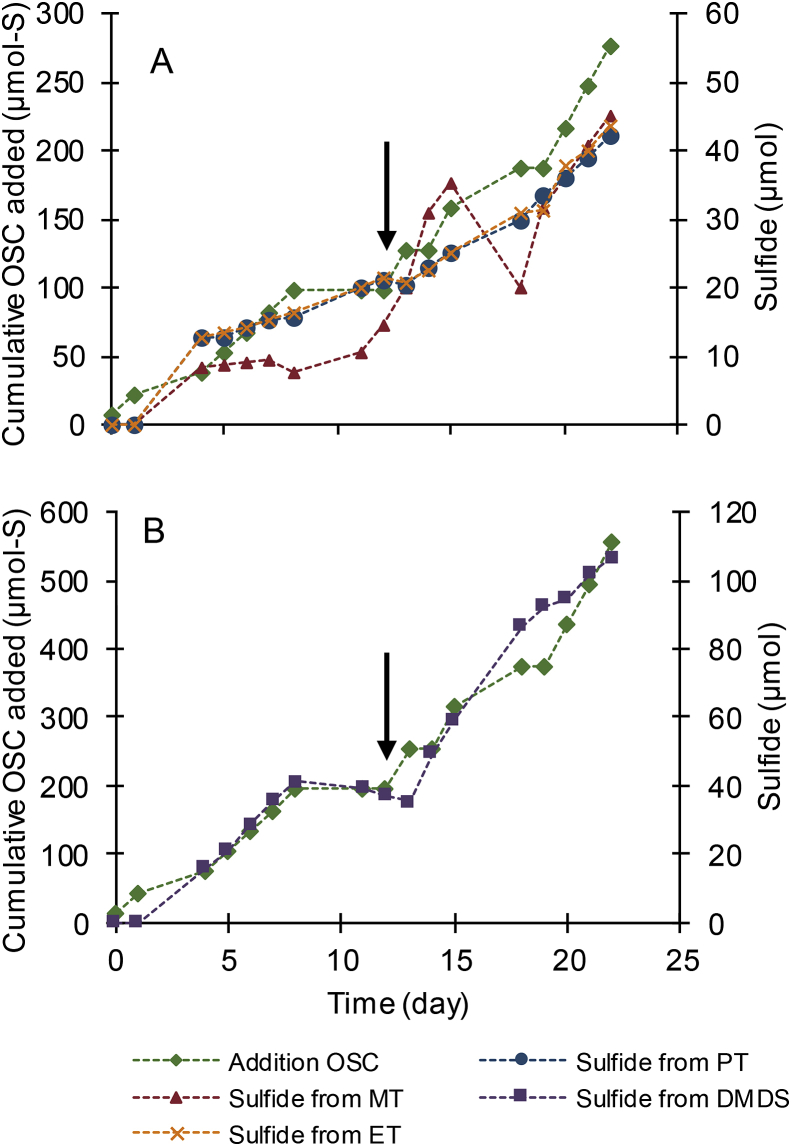
Each organosulfur compound was converted into sulfide. Cumulative organosulfur (OSC) additions expressed as μmol S and sulfide measured in the liquid phase in cells with **(A)** methanethiol (MT), ethanethiol (ET), propanethiol (PT) and **(B)** dimethyl disulfide (DMDS). Arrows indicate the day at which organosulfur additions were increased from 15 ​μmol to 30 ​μmol and the increase of current density from 2mA to 4mA.

Addition of organosulfur compounds over 22 days resulted in a total addition of 278 ​μmol (1.9 ​mM) sulfur for methanethiol, ethanethiol and propanethiol (Fig. 2a) and 555 ​μmol (3.7 ​mM) sulfur for dimethyl disulfide (Fig. 2b). Each biocathode, operating with different organosulfur compounds, showed a similar trend in the formation of sulfide. Sulfide was accumulating to 45 ​μmol (0.30 ​mM) for methanethiol, 44 ​μmol (0.29 ​mM) for ethanethiol, 42 ​μmol (0.28 ​mM) for propanethiol and 107 ​μmol (0.71 ​mM) for dimethyl disulfide during the experiment. The formation of sulfide in each of the biocathodes demonstrates that all organosulfur compounds were converted. Sulfide formation showed a faster increase with time after the current density and organosulfur compound additions were doubled, indicating that organosulfur compounds reduction continued at higher rates. The increased levels of organosulfur compounds did not inhibit the microbial community as sulfide production continued at an increased rate.

Sulfur recoveries as sulfide were 18% for methanethiol, 18% for ethanethiol, 17% for propanethiol and 22% for dimethyl disulfide compared to total added sulfur in the form of organosulfur compounds. For methanethiol, this recovery is lower than the sulfur recovery obtained in the initial 5 day experiment with methanethiol (section 3.1), in which recoveries of 41% and 64% were obtained. It is likely that this difference in sulfur recovery is related to the difference in operation and experimental run time. For example, the longer experimental run (22 days) received 11 additions of methanethiol, whereas the 5 day experimental run received only one addition. A more detailed study to close sulfur balances is needed in future studies.

17% of the sulfur added in the form of methanethiol was detected as sulfate, while sulfate recovery remained lower than 5% in the experiments with other organosulfur compounds. The higher sulfate production for the methanethiol fed biocathode might be a result of some oxygen intrusion found at this cathode (See SI-3). The presence of oxygen in this cell may have influenced the conversion mechanisms, as strictly anaerobic conditions could not be maintained. However, with the measured cathode potentials lower than −1 ​V vs. Ag/AgCl, it is expected that oxygen was quickly reduced. The formation of methane within the cell indicates that anaerobic processes were still in place.

The cathode potentials in all tests were ranging between −1.15 to −1.43 ​V vs. Ag/AgCl and slowly decreased throughout the experiment, indicating that hydrogen formation took place. The main element of the medium consists of a carbonate/bicarbonate buffer. Under the operating conditions, methane can be formed biologically from this inorganic carbon source. Hydrogen and methane were also detected in the gas phase (SI-3).

The coulombic efficiency (part of the total charge used for organosulfur compound reduction) was 1.7% for methanethiol, 3.4% for ethanethiol and 5.0% for propanethiol and dimethyl disulfide, under the assumption that the available organosulfur compounds were completely reduced towards methane and sulfide (See eq (1), (2), (3), (4))). In our experiments, we did observe methane formation, but it is also possible that other products rather than methane, e.g. CO_2_, ethane or propane were formed (not included for calculation of electron efficiency). Other electron sinks in this process were hydrogen and methane (See SI-3), and growth of biomass (not quantified). The effect of thiol concentration and current density on thiol degradation was not further studied in this manuscript and will be topic of further research.

### 3.3 Organosulfur degradation requires a combination of electricity and microorganisms

Electrochemical control experiments were performed to ensure that the degradation of organosulfur compounds was not merely a result of the applied current. The experiments showed no sulfide formation after 24 ​h of operation and indicate that microbial activity is required for the conversion of the tested organosulfur compounds. Cathode potentials during the control experiments ranged between −1.10 and −1.34 ​V vs. Ag/AgCl, similar to the cathode potential in the bioelectrochemical runs.

During the bioelectrochemical experiments, hydrogen was produced at the cathode, which could be used as alternative electron donor for organosulfur compound degradation. Therefore, biotic control experiments were performed with 20% hydrogen in the gas phase, without electrodes. Surprisingly, during 30 days, no sulfide was detected, demonstrating that both current and biomass are required for the degradation of the organosulfur compounds. Even though bio-degradation of methanethiol [[5], [6], [7], [8],^23^,^24^] and dimethyl disulfide [[6], [7], [8]] in anaerobic environments has been frequently reported, this was not shown in our control experiments, and may be the result of different conditions, such as halo-alkaline medium, selected inocula and experimental run time. Oremland et al. studied the degradation of ethanethiol in the presence and absence of hydrogen, the obtained results were contradictory and showed both increased and decreased degradation under the presence of hydrogen [9]. The exact role of electrical current and potentially hydrogen in organosulfur degradation is still unclear and needs to be elucidated in further research.

### 3.4 Microbial composition in organosulfur compound degrading BES

**Fig. 3:**
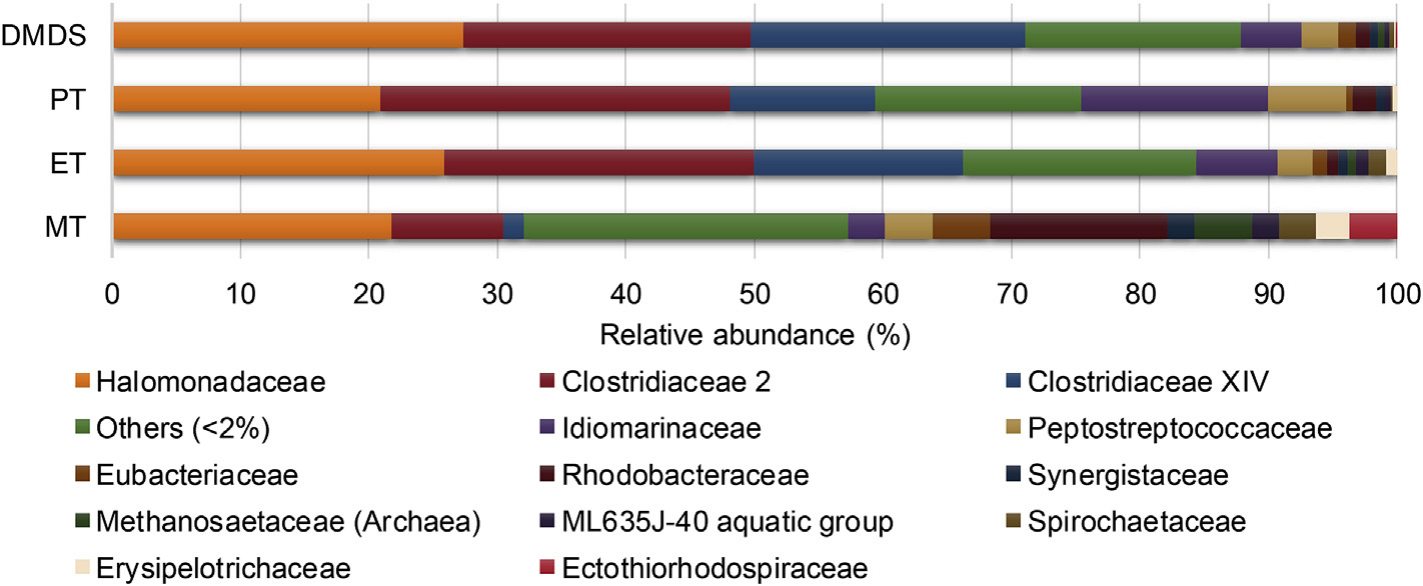
Relative abundance of the microbial community for cells with methanethiol (MT), ethanethiol (ET), propanethiol (PT) and dimethyl disulfide (DMDS) based on 16S rRNA sequencing. A similar microbial composition was found in all cells with dominant species belonging to the families *Halomonadaceae, Clostridiaceae* families *2 XIV*. Families with an abundancy <2% are summarized and shown as others.

Microbial community analyses were performed on the bioelectrodes collected at the end of the experiment (Section 3.2). A large similarity was found between cathodes fed with ethanethiol, propanethiol and dimethyl disulfide (Fig. 3). Dominant families on the cathodes were *Halomonadaceae*, *Clostridiaceae* families *2* and *XIV*. The cathode fed with methanethiol showed a lower abundancy for the two families *Clostridiaceae 2* and *XIV,* while the presence of *Rhodobacteraceae* was increased. The difference in microbial community on this cathode compared to the others potentially resulted from oxygen intrusion into this cell (See SI-3). The members of the families *Halomonodaceae* and *Clostridiaceae 2,* dominant in all cells, were also observed in a haloalkaline sulfide oxidizing bioreactor in the presence of methanethiol, ethanethiol and propanethiol [25]. *Clostridium*, one of the genera within the family of *Clostridiaceae*, is well known to be electroactive [26]. Since a mixture of inocula were used to study organosulfur degradation, experiments were performed in the same bioelectrochemical test setup to evaluate whether each separate inoculum had the capacity to degrade organosulfur compounds. Here, dimethyl disulfide was used. The cell inoculated with a mix of paper industry sludge and municipal wastewater treatment plant sludge showed sulfide formation after 4 days. Sludge from the chain elongation reactor showed sulfide formation after 5 days, and, sludge from the high salinity reactor and sludge adapted to the presence of organosulfur compounds both showed sulfide formation after 18 days. Degradation of dimethyl disulfide was thus possible by all inoculum sources separately.

### 3.5 Outlook

Thiols and hydrogen sulfide are often both present in gaseous waste streams. Biodesulfurization technologies focused on the recovery of elemental sulfur from hydrogen sulfide suffer from the presence of organosulfur compounds [27]. The conversion of these organosulfur compounds towards hydrogen sulfide would in these cases be an advantage as this can be detoxified together with the hydrogen sulfide and be removed in existing, well-known efficient treatment plants.

This research presents a proof of principle for the reduction of methanethiol, ethanethiol propanethiol and dimethyl disulfide at biocathodes towards sulfide. Various aspects need to be considered to further study and develop this new application of BES. Coulombic efficiencies in this experimental design were low and can be improved by limiting (i) the formation of methane resulting from carbon dioxide reduction and (ii) hydrogen formation at the electrode. When calculating the coulombic efficiency, we assumed a complete reduction towards methane, however, metabolic pathways and products were not further identified. Further design questions involve defining reaction kinetics, microbial growth rates, evaluating long-term process stability and the role of methanol as co-substrate. The economic feasibility of this system will largely depend on maximum attainable reduction rates and microbial toxicity limits. Regardless the remaining research questions, this application of BES demonstrated a new potential strategy to biologically convert organosulfur to sulfide, a product for which many efficient sulfur recovery technologies are available.

## Declaration of competing interest

The authors declare that they have no known competing financial interests or personal relationships that could have appeared to influence the work reported in this paper.

## Appendix A Supplementary data

Supplementry data Supplementry data

## References

[1] Lee C.L., Brimblecombe P. (2016). Anthropogenic contributions to global carbonyl sulfide, carbon disulfide and organosulfides fluxes. Earth Sci. Rev..

[2] Bentley R., Chasteen T.G. (2004). Environmental VOSCs - formation and degradation of dimethyl sulfide, methanethiol and related materials. Chemosphere.

[3] De Angelis A. (2012). Natural gas removal of hydrogen sulphide and mercaptans. Appl. Catal. B Environ..

[4] Ramírez M., Fernández M., Granada C., Le Borgne S., Gómez J.M., Cantero D. (2011). Biofiltration of reduced sulphur compounds and community analysis of sulphur-oxidizing bacteria. Bioresour. Technol..

[5] De Bok F.A.M., Van Leerdam R.C., Lomans B.P., Smidt H., Lens P.N.L., Janssen A.J.H., Stams A.J.M. (2006). Degradation of methanethiol by methylotrophic methanogenic archaea in a lab-scale upflow anaerobic sludge blanket reactor. Appl. Environ. Microbiol..

[6] Kiene R.P., Oremland R.S., Cantena A., Miller L.G., Capone D.G. (1986). Metabolism of reduced methylated sulfur compounds in anaerobic sediments and by a pure culture of an estuarine methanogent. Appl. Environ. Microbiol..

[7] Sipma J., van Bree R., Hulshoff Pol L.W., Janssen A.J.H., Lettinga G., Arena B. (2006). Degradation of methanethiol in a continuously operated upflow anaerobic sludge-blanket reactor. Water Environ. Res..

[8] Van Leerdam R.C., De Bok F.A.M., Lomans B.P., Stams A.J.M., Lens P.N.L., Janssen A.J.H. (2006). Volatile organic sulfur compounds in anaerobic sludge and sediments: biodegradation and toxicity. Environ. Toxicol. Chem..

[9] Oremland R.S., Whiticar M.J., Strohmaier F.E., Kiene R.P. (1988). Bacterial ethane formation from reduced, ethylated sulfur compounds in anoxic sediments. Geochem. Cosmochim. Acta.

[10] Xie S., Lazar C.S., Lin Y.S., Teske A., Hinrichs K.U. (2013). Ethane- and propane-producing potential and molecular characterization of an ethanogenic enrichment in an anoxic estuarine sediment. Org. Geochem..

[11] Zamora P., Georgieva T., Ter Heijne A., Sleutels T.H.J.A., Jeremiasse A.W., Saakes M., Buisman C.J.N., Kuntke P. (2017). Ammonia recovery from urine in a scaled-up microbial electrolysis cell. J. Power Sources.

[12] Liu D., Zhang L., Chen S., Buisman C., Ter Heijne A. (2016). Bioelectrochemical enhancement of methane production in low temperature anaerobic digestion at 10 °C. Water Res..

[13] Wang H., Ren Z.J. (2014). Bioelectrochemical metal recovery from wastewater: a review. Water Res..

[14] Geppert F., Liu D., van Eerten-Jansen M., Weidner E., Buisman C., ter Heijne A. (2016). Bioelectrochemical power-to-gas: state of the art and future perspectives. Trends Biotechnol..

[15] Logan B.E., Hamelers B., Rozendal R., Schröder U., Keller J., Freguia S., Aelterman P., Verstraete W., Rabaey K. (2006). Microbial fuel cells: methodology and technology. Environ. Sci. Technol..

[16] de Rink R., Klok J.B.M., van Heeringen G.J., Sorokin D.Y., ter Heijne A., Zeijlmaker R., Mos Y.M., de Wilde V., Keesman K.J., Buisman C.J.N. (2019). Increasing the selectivity for sulfur formation in biological gas desulfurization. Environ. Sci. Technol..

[17] de Smit S.M., de Leeuw K.D., Buisman C.J.N., Strik D.P.B.T.B. (2019). Continuous n-valerate formation from propionate and methanol in an anaerobic chain elongation open-culture bioreactor. Biotechnol. Biofuels.

[18] Sudmalis D., Gagliano M.C., Pei R., Grolle K., Plugge C.M., Rijnaarts H.H.M., Zeeman G., Temmink H. (2018). Fast anaerobic sludge granulation at elevated salinity. Water Res..

[19] Sousa J.A.B., Jánoska A., Bijmans M.F.M., Stams A.J.M., Plugge C.M. (2015). Dimethyldisulfide degradation by anaerobic microorganisms at haloalkaline conditions. EMBO Work. Microb. Sulfur Metab.

[20] Roman P., Veltman R., Bijmans M.F.M., Keesman K.J., Janssen A.J.H. (2015). Effect of methanethiol concentration on sulfur production in biological desulfurization systems under haloalkaline conditions. Environ. Sci. Technol..

[21] Liu D., Zheng T., Buisman C., Ter Heijne A. (2017). Heat-treated stainless steel felt as a new cathode material in a methane-producing bioelectrochemical system. ACS Sustain. Chem. Eng..

[22] Takahashi S., Tomita J., Nishioka K., Hisada T., Nishijima M. (2014).

[23] Sipma J., Janssen A.J.H., Hulshoff Pol Look W L.W.H., Lettinga G. (2003). Development of a novel process for the biological conversion of H2S and methanethiol to elemental sulfur. Biotechnol. Bioeng..

[24] Yuan Z., Sharma K.R., Hu S., Ni B.-J., Sun J. (2014). Degradation of methanethiol in anaerobic sewers and its correlation with methanogenic activities. Water Res..

[25] Roman P., Klok J.B.M., Sousa J.A.B., Broman E., Dopson M., Van Zessen E., Bijmans M.F.M., Sorokin D.Y., Janssen A.J.H. (2016). Selection and application of sulfide oxidizing microorganisms able to withstand thiols in gas biodesulfurization systems. Environ. Sci. Technol..

[26] Koch C., Harnisch F. (2016). Is there a specific ecological niche for electroactive microorganisms?. ChemElectroChem.

[27] Roman P., Bijmans M.F.M., Janssen A.J.H. (2016). Influence of methanethiol on biological sulphide oxidation in gas treatment system. Environ. Technol..

