# Supplementary material for: Microbial reduction of organosulfur compounds at cathodes in bioelectrochemical systems
Source: Environ Sci Ecotechnol. 2020 Jan 7;1:100009. doi: 10.1016/j.ese.2020.100009 (PMC9488095; doi:10.1016/j.ese.2020.100009)
Supplement: Supplementry data [file mmc1.pdf]

Supporting information

Microbial reduction of organosulfur compounds at cathodes in bioelectrochemical systems

Margo Elzinga[1,2], Dandan Liu[1,2], Johannes B.M. Klok[1,2,3], Pawel Roman[3], Cees. J. N. Buisman[1,3], Annemiek ter Heijne[1*]

1 Environmental Technology, Wageningen University, Bornse Weilanden 9, P.O. Box 17, 6700 AA Wageningen, The Netherlands

2 Paqell B.V., Reactorweg 301, 3542 AD Utrecht, The Netherlands

3 Wetsus, Centre of Excellence for Sustainable Water Technology, Oostergoweg 9, P.O. Box 1113, 8900 CC Leeuwarden, The Netherlands

Contents:

SI-1 Ion chromatography method

SI-2 OSC headspace concentrations in abiotic control of methanethiol degradation in BES

SI-3 Gas phase composition and coulombic efficiency for degradation of organosulfur compounds

SI-1 Ion chromatography method

Ion chromatography (Dionex ICS 2100 RFIC, Salt Lake City, USA) was used to determine the concentrations of sulphate and thiosulphate. An IonPac AS17C column 250x4mm was used at 30C with a flow rate of 1.0 ml per minute. The high carbonates were bypassed with a carbonate trap. The injection volume was 10µL. The eluent was generated by an eluent generator (EG40, Dionex, Salt Lake City, USA) equipped with a KOH cartridge, and carried by deionized water. Detection of the ions was based on conductivity; we used an ADRES 600 4mm suppressor to suppress eluent conductivity.

SI-2 OSC headspace concentrations in abiotic control of methanethiol degradation in BES

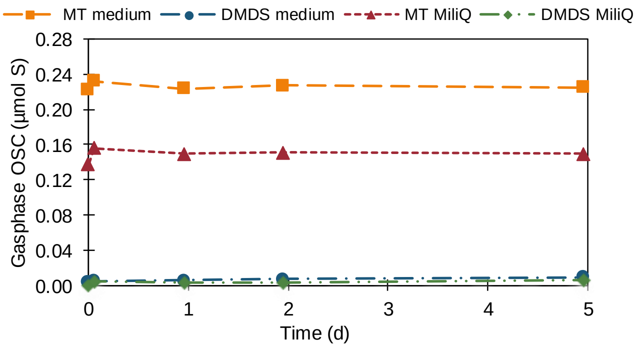

Figure SI-2 Organosulfur compounds (OSC) present as methanethiol (MT) and its oxidation product dimethyl disulfide (DMDS)in the gas phase of anaerobic abiotic control experiments performed in medium and Milli-Q.

SI-3 Gas phase composition coulombic efficiency CEs for degradation of organosulfur compounds experiment

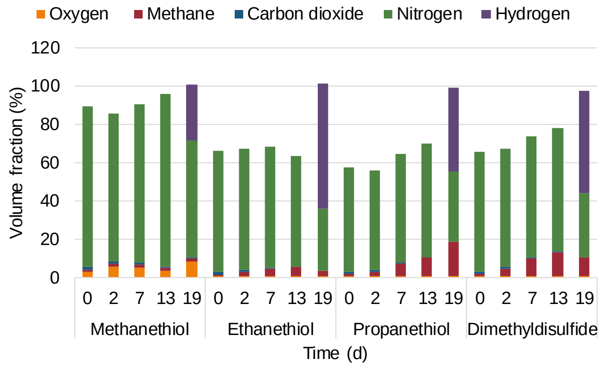

Figure SI-3 Gas phase composition of cells fed with methanethiol, ethanethiol, propanethiol and dimethyl disulfude. Oxygen found in the methanethiol-fed cells are higher compared to the other cells and indicate oxygen intrusion into the system. Hydrogen was only measured at day 19.

Hydrogen sulfide measurements were only performed at day 19 of the experiment. Since we have measured all other main components in the gas phase, we can make a rough calculation how much hydrogen would be present the other days. If we assume that the main components are methane, carbon dioxide, nitrogen and oxygen, hydrogen will be the remaining fraction of the headspace. Another issue is that we measured gas volumes only at the beginning and the end of the experiment, so we would need to assume a linear increase in gas production. We calculated the coulombic efficiency (CE) with these assumptions, and this shows that hydrogen was indeed one of the main electron sinks. The CE of these products are shown in table SI-3.

Table SI-3 Coulombic efficiencies

Cell operated with	Methane*	Hydrogen	Total
Methanethiol	4.7	24.9	29.6
Ethanethiol	9.9	56.0	65.9
Propanethiol	61.4	37.6	99.0
Dimethyldisulfide	33.2	45.8	79.0

* We assume reduction of carbon dioxide to methane which requires 8 electrons.
